# Supplementary material for: Hospital Antibiotic Use during COVID-19 Pandemic in Italy
Source: Antibiotics (Basel). 2023 Jan 13;12(1):168. doi: 10.3390/antibiotics12010168 (PMC9854455; doi:10.3390/antibiotics12010168)

Hospital Antibiotic Use during COVID-19 Pandemic in Italy

Alessandro Perrella [1,*], Filomena Fortinguerra [2,*], Andrea Pierantozzi [2], Nicolina Capoluongo [1], Novella Carannante [1], Andrea Lo Vecchio [3], Francesca Futura Bernardi [4], Francesco Trotta [2] and Agnese Cangini [2]

[1] I Division Emerging Infectious Disease and High Contagiousness, D. Cotugno Hospital, Naples, Italy
[2] Italian Medicines Agency (AIFA), Rome, Italy
[3] Pediatric Unit, AOU Federico II Medical School University, Naples, Italy
[4] Pharmacy Unit, Vanvitelli University, Naples, Italy
* Correspondence: alessandro.perrella@ospedalideicolli.it (A.P.); f.fortinguerra@aifa.gov.it (F.F.)

Supplementary Materials

Figure S1 Correlation between consumption (DDD) of antibiotics for systemic use (J01) in the hospital setting and number of hospitalisation with COVID-19 symptoms and in intensive care in 2020 *(r di Pearson* hospitalisation with COVID-19 symptoms*: 0.82; r di Pearson intensive care: 0.87)*

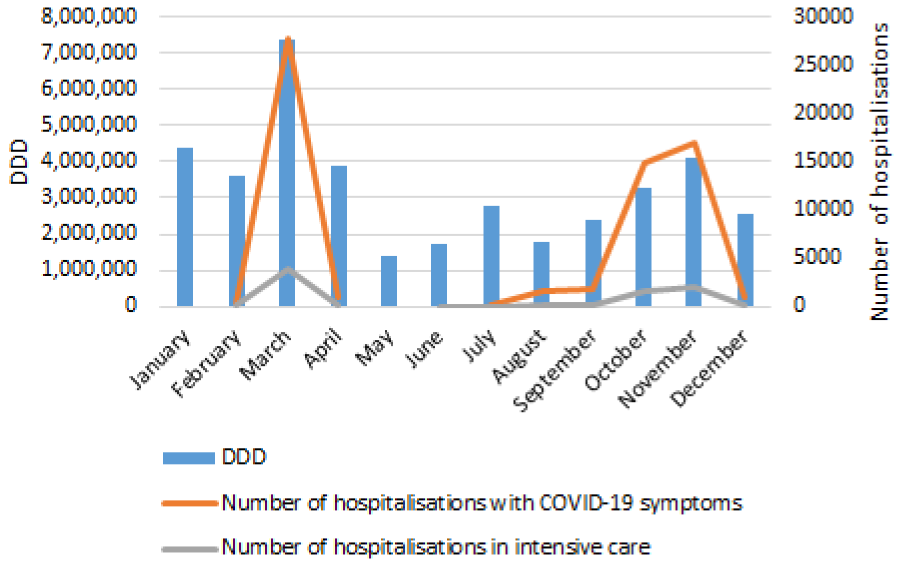

Figure S2 Correlation between consumption (DDD) of third generation cephalosporins (J01DD) in the hospital setting and number of hospitalisations with COVID-19 symptoms and in intensive care in 2020 *(r di Pearson* hospitalisations with COVID-19 symptoms*: 0.88; r di Pearson intensive care: 0.91)*

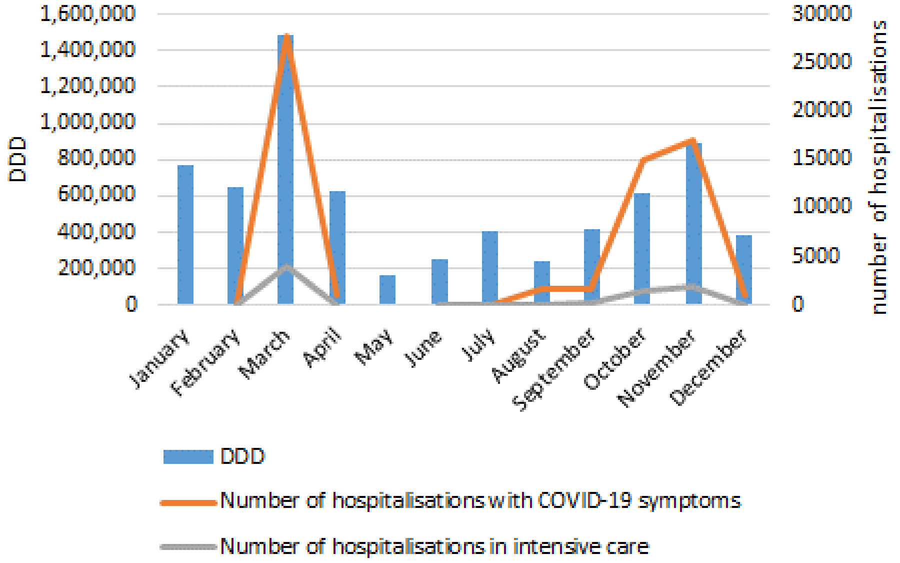

Figure S3 Correlation between consumption (DDD) of carbapenems (J01DH) in the hospital setting and number of hospitalisations with COVID-19 symptoms and in intensive care in 2020 *(r di Pearson hospitalisations with COVID-19 symptoms: 0.16; r di Pearson intensive care: 0.29)*

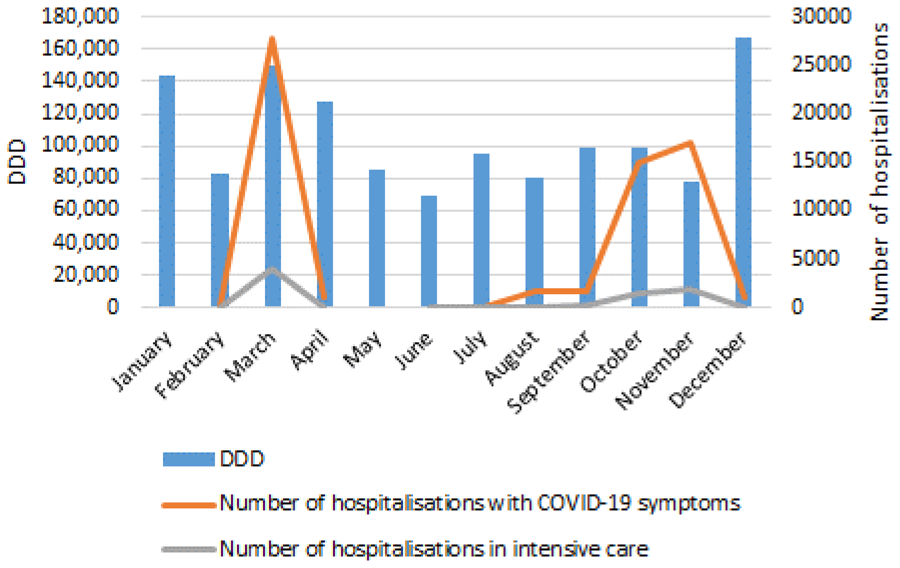

Figure S4 Correlation between consumption (DDD) of macrolides (J01FA) in the hospital setting and number of hospitalisations with COVID-19 symptoms and in intensive care in 2020 *(r di Pearson hospitalisations with COVID-19 symptoms: 0.73; r di Pearson intensive care: 0.79)*

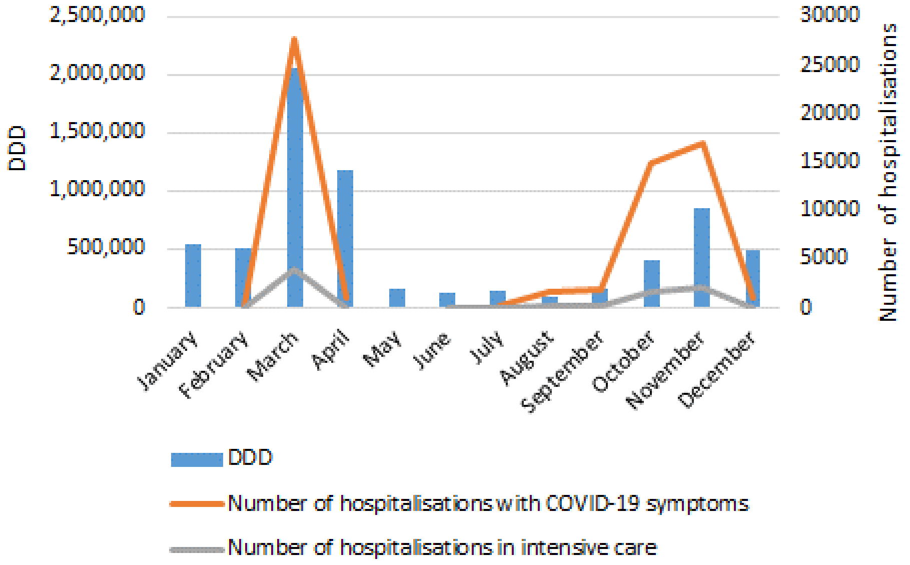

Figure S5 Correlation between consumption (DDD) of fluoroquinolones (J01MA) in the hospital setting and number of hospitalisations with COVID-19 symptoms and in intensive care in 2020 *(r di Pearson hospitalisations with COVID-19 symptoms: 0.73; r di Pearson intensive care: 0.79)*

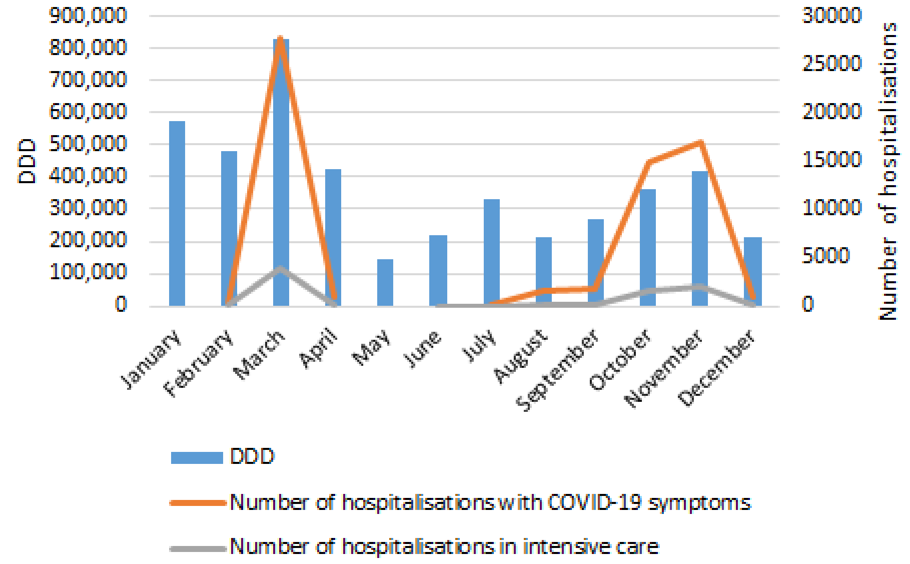

Supplement: Supplementary file 1 [file antibiotics-12-00168-s001.zip › antibiotics-2141448-supplementary.pdf]
